# Vitamin D intake of Dutch infants from the combination of (fortified) foods, infant formula, and dietary supplements

**DOI:** 10.1007/s00394-015-1102-z

**Published:** 2015-11-24

**Authors:** Janneke Verkaik-Kloosterman, Marja H. Beukers, Martine Jansen-van der Vliet, Marga C. Ocké

**Affiliations:** National Institute for Public Health and the Environment, PO Box 1, 3720 BA Bilthoven, The Netherlands

**Keywords:** Vitamin D, Infant, Excessive intake, Food, Supplements, Infant formula

## Abstract

**Purpose:**

Due to changes in the Dutch fortification policy for vitamin D and the vitamin D supplementation advice for infants (10-μg/d for 0–4 year olds), a partially virtual scenario study was conducted to evaluate the risk of excessive vitamin D intake assigning all infants to a 100 % adherence to the supplementation advice and considering the current fortification practice.

**Methods:**

Food consumption data from the Nutrition Intake Study (2002; *N* = 941, 7–19 months) were combined with Dutch food composition data from 2011 to estimate vitamin D intake from (fortified) foods. For infants 0–6 months of age, the consumption volume infant formula was estimated from energy requirement and body weight. All subjects were assigned to take a daily 10 µg vitamin D supplement, according the Dutch supplementation advice for infants. Habitual vitamin D intake was estimated using the Statistical Program to Assess Dietary Exposure and compared with the tolerable upper intake levels (ULs) set by the European Food Safety Authority.

**Results:**

The median habitual total vitamin D intake was 16–22 µg/day for infants aged 0–6 months (increasing with age) and 13–21 µg/day for infants aged 7–19 months (decreasing with age). About 4–12 % of infants aged 7–11 months exceeded the UL. At the 99th percentile, the intake was 2–4 µg above the UL, depending on age. Infants aged 0–6 and 12–19 months did not exceed the UL.

**Conclusions:**

In case of combined intake from infant formula, (fortified) foods, and supplements, vitamin D intakes above the UL are possible among some infants during a limited time period.

## Introduction

Vitamin D is important for the development of strong bones. Serious vitamin D deficiency in infants leads to rickets [1, 2]. The Health Council of the Netherlands set the adequate intake (AI) for infants at 10 µg/day (i.e. 400 IU/day) [1]. Too high vitamin D intake, on the other hand, should be prevented as this may result in risk of hypercalcaemia or hypercalciuria and kidney problems. Both the European Food Safety Authority (EFSA) and the Institute of Medicine (IoM) set a tolerable upper intake level (UL) for vitamin D [2, 3]. For infants 0–6 months of age, the UL is set at 25 µg/day by both EFSA and IoM, and for older infants (6–12 months), the UL is set at 25 µg/day by EFSA and 37.5 µg/day by IoM [2, 3]. The UL for children aged 1 year and older is set at 50 µg/day by EFSA and at 63 µg/day by the IoM [2, 3].

Vitamin D can be synthesised in the skin by exposure to sunlight. The actual production depends on many factors including latitude, season, time of the day, and exposure duration. In the Netherlands at 52° northern latitude, vitamin D production by the skin is possible between March and November at moments the zenith angle is not oblique (i.e. >45°) [1, 4, 5]. However, infants should be protected from direct sunlight exposure to avoid sunburn and reduce the risk of skin cancer in later life [6]. Consequently, infants rely mostly on their diet for an adequate vitamin D supply.

Vitamin D is naturally present in foods. In addition, vitamin D may be added to foods like infant or follow-on formula [7]. There is a history of addition of vitamin D to margarines and baking fats (covenant between Ministry of Health and manufacturers [8, 9]), generally with a level of 7.5 µg/100 g. And since 2007, voluntary fortification of other foods is permitted in the Netherlands up to a maximum of 4.5 µg/100 kcal [10, 11].

Similar to other countries (e.g. [12, 13]), the Dutch Health Council advices vitamin D supplementation for children up to 4 years of age [14], in order to compensate for the minimal exposure to sunlight and generally limited vitamin D levels in food. Since 2008, the advice was to give daily a supplement with 10 µg vitamin D for all young children (0–4 years), except those consuming more than 500 ml infant formula a day [14]. To improve the use of adequate vitamin D supplementation in the group of infants transferring from infant formula to regular milk, the supplementation advice was revised in 2012 to 10 µg/day for all children up to 4 years of age, irrespective of their diet [1].

The vitamin D intake of infants may aggregate from different sources, and as such, infants may be at risk of too high vitamin D intakes. The objective of this study was to estimate the total vitamin D intake of infants for the scenario that all infants adhere to the current supplementation advice and the current practice regarding food fortification, and to investigate the risk of excessive intakes.

## Subjects and methods

### Food consumption data

#### Infants aged 0 until 6 months

For infants aged 0 until 6 months, no food consumption data are available in the Netherlands. Therefore, for infants of this age an estimation of the consumption volume of infant formula was made based on energy requirement [15] and body weight from the Dutch growth study [16]. The energy requirement was set at 0.39 MJ per kg body weight for infants aged 0–2 months and at 0.35 MJ per kg body weight for infants aged 3–5 months. The median and 97.5th percentile of the body weight distribution of Dutch infants of different ages (Table 1) were combined with these energy requirements to estimate the energy requirement in MJ/d as an indicator of the energy intake per day as a proxy for children with median and high energy intake. The legal range of energy content of infant formula is 60–70 kcal/100 ml [17]. In this study, the midpoint of 65 kcal/100 ml was used to estimate the consumption volume of infant formula per day (ml/day) from the estimated daily energy intake.

**Table 1:** Estimation of the vitamin D intake (µg/day) for Dutch infants aged 0–6 months based on energy requirement [15] and body weight (bw) [16], including and excluding the advice of a daily supplement (suppl) containing 10 µg vitamin D [1]

Age (months)	Energy requirement (kcal/kg bw)	Body weight (kg)		Energy intake (kcal/d)		Vitamin D intake (µg/day)			
						P50 bw		P97.5 bw	
		P50	P97.5	P50	P97.5		+10 µg from suppl		+10 µg from suppl
*Boys*									
0–1	93	3.9	4.9	363	456	6.7	16.7	8.4	18.4
1–2	93	4.7	5.9	438	550	8.1	18.1	10.1	20.1
2–3	84	5.5	6.8	460	568	8.5	18.5	10.5	20.5
3–4	84	6.2	7.7	518	644	9.6	19.6	11.9	21.9
4–5	84	7.1	8.8	594	736	11.0	21.0	13.6	23.6
5–6	84	7.6	9.5	635	794	11.7	21.7	14.7	24.7
*Girls*									
0–1	93	3.7	4.6	345	428	6.4	16.4	7.9	17.9
1–2	93	4.4	5.5	410	512	7.6	17.6	9.5	19.5
2–3	84	5.1	6.3	426	527	7.9	17.9	9.7	19.7
3–4	84	5.7	7.1	476	594	8.8	18.8	11.0	21.0
4–5	84	6.6	8.2	552	685	10.2	20.2	12.7	22.7
5–6	84	7.1	8.8	594	736	11.0	21.0	13.6	23.6

#### Infants aged 7–19 months

Data of the Nutrient Intake Study (VIO study) were used as source of the consumed foods of Dutch infants. This survey is the most recent Dutch survey among infants aged 7–19 months (*N* = 941, response rate 82.5 %) and has been described in detail elsewhere [18, 19]. Briefly, data were collected between January and June 2002 among a representative sample of Dutch infants. Healthy, non-breastfed, term-born infants (37–41 weeks of pregnancy) with a birth weight of at least 2500 g were recruited via 33 Dutch child health centres (circa 95 % of children (0–4 years) visit these centres; between 0 and 1 year, this proportion is even higher [20]), in three age groups: 9 (7–10) months, 12 (11–13) months, and 18 (17–19) months (*n* = 333, *n* = 306, *n* = 302, respectively). Data on food consumption were collected on two non-consecutive days (preferably on 1 week and one weekend day) by a diary filled in by the parents or caretakers. Several household measures (e.g. teaspoon, measuring jug) were supplied to assist the reporting of the consumed amounts. To be able to estimate the nutrient intake, the consumed foods were coded according to the Dutch food composition database 2001 [21].

### Scenario

Due to the changed Dutch fortification policy for vitamin D and the changed supplementation advice for infants, a scenario was designed to estimate the potential total vitamin D intake. In this scenario (a), the vitamin D intake from food was estimated for the 2011 situation applying the 2011 fortification practice to the 2002 food consumption data (see below). In an additional scenario (b), next to vitamin D intake from foods (scenario a), all infants were assigned to a 100 % adherence to the use of a daily supplement containing 10 µg vitamin D conforming the supplementation advice for infants in the Netherlands.

#### Food composition

End 2011 we made an inventory of vitamin D fortified foods intended for young children (0–4 years) available on the Dutch market. For this inventory, information available in the Netherlands Food Information Resource (NethFIR) containing the Dutch food composition database (NEVO; using the databases of 2006 and 2011) [22, 23] and a web application for brand foods (www.levensmiddelendatabank.nl) was used. Also, a supermarket inventory was held and searches were done on manufacturer’s websites and the INNOVA database (www.innovadatabase.com). The total vitamin D content as labelled was collected for all vitamin D fortified foods.

Originally, the food consumption data from VIO were combined with Dutch food composition data from 2001 [21]. However, to get insight into the current vitamin D intake, including the current fortification practice, for this study the food consumption data were combined with Dutch food composition data from 2011 (www.rivm.nl/nevo) supplemented with the results from the inventory. For foods coded with a code no longer present in 2011, the vitamin D content of a similar food was used. If there were more possibilities for similar foods, the one with the highest vitamin D content was selected. More than 75 % of the food codes were replaced with more recent data. If no appropriate replacing food was available, the vitamin D content of 2001 was retained. These were mainly foods from food groups containing no or very low vitamin D levels in both 2001 and 2011, e.g. infant fruit meal, syrup.

As there was only little variation in the vitamin D content of infant formula, for infants aged 0–6 months the mean vitamin D content in infant formula from the inventory of end 2011 was used and multiplied with the estimated consumption volume of infant formula.

### Statistical analyses

To estimate the vitamin D intake for infants 0–6 months of age, the volume of infant formula estimated based on energy requirement and median or 97.5th percentile of body weight distribution was multiplied with the vitamin D content of infant formula. It was assumed that these infants did not consume complementary foods. The estimated vitamin D intake was compared to the UL of 25 µg/day as set by EFSA [3] to assess the risk of excessive intake. For infants 7–19 months of age, the vitamin D intake was calculated by multiplying the consumed amounts of foods with the vitamin D content of these foods, resulting in a total vitamin D intake per subject for each study day. The consumption data were collected over a short-term period (day), and the individual consumption can vary considerably from day to day (i.e. within-person variation). Consequently, without correction for within-person variation, intake measured on a limited number of days per individual will be a poor indicator of the population habitual intake distribution (also referred to as usual intake). The use of such data in the evaluation of the risk of excessive intake may give invalid results. Therefore, the data in our study were statistically corrected for within-person variation to get the habitual vitamin D intake distribution using the Statistical Program to Assess Dietary Exposure (SPADE version 2.25) [24, 25].

In the VIO study, most children consumed infant or follow-on formula on both study days or did not consume it at all. As vitamin D is added to infant and follow-on formula, it contributed largely to total vitamin D intake of consumers of these products. Consequently, the vitamin D intake distribution was multimodal. In the estimation of the habitual intake, it is assumed that the intake can be transformed to a more or less normal distribution, and this was not the case for the multimodal distribution. It was therefore not appropriate to estimate the habitual vitamin D intake distribution from the total vitamin D intake per day [19]. We therefore applied a so-called first-shrink-then-add approach [26–28] and estimated the habitual intake distribution separately for vitamin D from infant/follow-on formula and for vitamin D from other food sources. For vitamin D from other food sources, a 1-part model was applied, as this was consumed daily by all subjects. With a 1-part model, the habitual intake amount is modelled, not the intake frequency, as all subjects have a daily intake. For vitamin D intake from infant/follow-on formula, a 2-part model was applied as part of the infants did not consume infant/follow-on formula on the studied days. These two habitual intake distributions were combined using Monte Carlo to obtain the habitual total vitamin D intake distribution.

For scenario b, all infants were assigned to take daily a dietary supplement containing 10 µg vitamin D. This amount was added to the vitamin D from other food sources, as it did not contain any within- or between-person variation. The estimation of the habitual total vitamin D intake distribution went via same procedure as described for scenario a.

To get insight into potential differences in habitual total vitamin D intake distributions for infants of different months of ages, the habitual intake was modelled as a function of age. This was performed separately for infants 7–13 and 16–19 months of age, because data on infants 14 and 15 months of age were lacking. Uncertainty in modelling the habitual intake was quantified with bootstrap analyses (1000 iterations) and is reported as 95 % confidence intervals around the point estimates.

The proportion of infants (7–19 months) with habitual total vitamin D intakes above the UL as set by EFSA (i.e. cut-off value of 25 µg/day for infants below 1 year and of 50 µg/day for infants 1 year and older [3]) was calculated.

## Results

### Inventory of vitamin D fortified foods

In 2011, vitamin D was voluntary added to some breakfast cereals, cookies, dairy products, and drinks (Table 2). The labelled vitamin D levels (total of natural vitamin D and added level) varied from 0.74 µg/100 g in soy drink to 16.5 µg/100 g in porridge cereals. In accordance with EU legislation [29], baby foods, other than cereal-based foods, were not fortified with vitamin D. Infant formula (based on 33 products) contained on average 1.2 µg (SD 0.2) vitamin D per 100 ml. Follow-on formula (based on 71 products) contained on average 1.4 µg (SD 1.0) vitamin D per 100 ml. For both types of formula, the levels were below the legal maximum amounts of 2.5 and 3.0 µg/100 kcal, respectively.

**Table 2:** Overview of foods voluntarily fortified with vitamin D (specifically for young children) on the Dutch market (end 2011), number of foods (number of brands) within different food groups and specification of types of foods that are fortified

Food group	No. of vitamin D fortified foods (no brand^a^)	Type of food	µg/100g^b^
Breakfast cereals	14 (3)	Porridge cereals	5–16.5
	7 (3)	Breakfast cereals	1.7–10
Cookies	3 (2)	Infant cookies	3–10
Dairy products	11 (6)	(Fruit) fromage frais	0.95–1.25
	11 (3)	Ready-to-drink milk porridge	1–2
	1 (1)	Yoghurt drink	0.75
	18 (6)	Toddler milk	0.9–2.1
Drinks	1 (1)	Instant cacao powder	7.1
	1 (1)	Soja drink junior	0.74

^a^The number between brackets is the number of different brands in which vitamin D fortified food products were identified

^b^Values are presented in the number of digits presented on the nutrition value declaration

### Vitamin D intake of infants aged 0–6 months

Based on energy requirement [15] and body weight reported in the fourth Dutch national growth study [16], an indication of the energy intake was provided for children 0–6 months of age. Taking the median body weight, these intakes varied from 345 kcal/day for girls aged 0–1 month to 635 kcal/day for boys aged 5–6 months (Table 1). This range was 428 kcal/day for girls aged 0–1 month to 794 kcal/day for boys aged 5–6 month when taking the 97.5th percentile of the body weight curve (Table 1). In 2011, the vitamin D level in infant formula intended for children aged 0–6 months was about 1.8 µg/100 kcal, based on label information. Taking this vitamin D level, the intake ranged from 6.4 µg/day for girls aged 0–1 month using median body weight to 14.7 µg/day for boys aged 5–6 months using 97.5th percentile of body weight. For the whole age group 0–6 months, estimated vitamin D intake from infant formula remained below the UL of 25 µg/day. Also with the addition of a daily supplement containing 10 µg vitamin D, intakes were below the UL. However, for children 4–6 months, considering the 97.5th percentile of body weight, vitamin D intake from infant formula and a dietary supplement was close to the UL, 22.7–24.7 µg/day (Table 1).

### Consumption of infant formula for infants aged 7–19 months

The percentage of infants 7–19 months of age consuming infant formula decreased with age (Table 3). About 95 % of the infants aged 7–9 months consumed infant formula, compared to 5–16 % of the infants 16–19 months of age. Most infants consuming infant formula did this on both study days. Besides the age-related decrease in the proportion of infants consuming infant formula, also the consumed volume decreased with age (Table 4). Among users of infant formula, the median habitual consumption of infant formula decreased from 592 ml/day for infants aged 7 months to 253 ml/day for infants aged 19 months.

**Table 3:** Users of follow-on formula^a^ in the Netherlands by age (*n* and % of total study population)

Age (months)	*N*	Users infant formula	
		***N***	**%**
7	19	18	95
8	117	112^b^	96
9	178	171^c^	96
10	48	44	92
11	193	144^d^	75
12	72	32^b^	44
13	12	4	33
16	26	3	12
17	88	14	16
18	150	19^e^	13
19	38	2	5

^a^This includes infant formula marketed for infants 0–6 months of age, which was consumed by small part of the infants

^b^One child one-day use

^c^Two children one-day use

^d^Eight children one-day use

^e^Four children one-day use

**Table 4:** Habitual intake distribution of follow-on formula^a^ (ml/day) for infants aged 7–13 and 16–19 months consuming infant formula on at least one study day, in the Netherlands. Presented as point estimate with between brackets 95 % CI

Age (months)	P5	P25	P50	P75	P95
7	348 (319–429)	490 (466–567)	592 (569–667)	695 (670–771)	846 (815–927)
8	315 (290–372)	457 (439–509)	558 (541–607)	660 (641–706)	810 (784–858)
9	283 (261–336)	424 (406–474)	524 (507–572)	626 (604–672)	775 (744–821)
10	251 (228–307)	391 (371–442)	490 (470–541)	592 (566–640)	741 (707–791)
11	220 (198–277)	358 (338–412)	457 (436–509)	558 (534–609)	706 (671–758)
12	189 (163–252)	325 (301–386)	424 (396–485)	524 (493–584)	671 (632–736)
13	159 (128–249)	293 (261–385)	391 (355–481)	490 (450–582)	637 (588–730)
16	165 (109–300)	291 (219–423)	398 (305–546)	519 (386–709)	718 (513–973)
17	132 (91–241)	247 (204–337)	346 (288–442)	461 (360–583)	650 (464–838)
18	103 (57–209)	205 (159–297)	298 (242–384)	405 (312–508)	586 (408–733)
19	77 (30–204)	168 (100–300)	253 (171–382)	353 (248–484)	524 (357–686)

^a^This includes infant formula marketed for infants 0–6 months of age, which was consumed by small part of the infants

### Vitamin D intake of infants aged 7–19 months

**Fig. 1:**
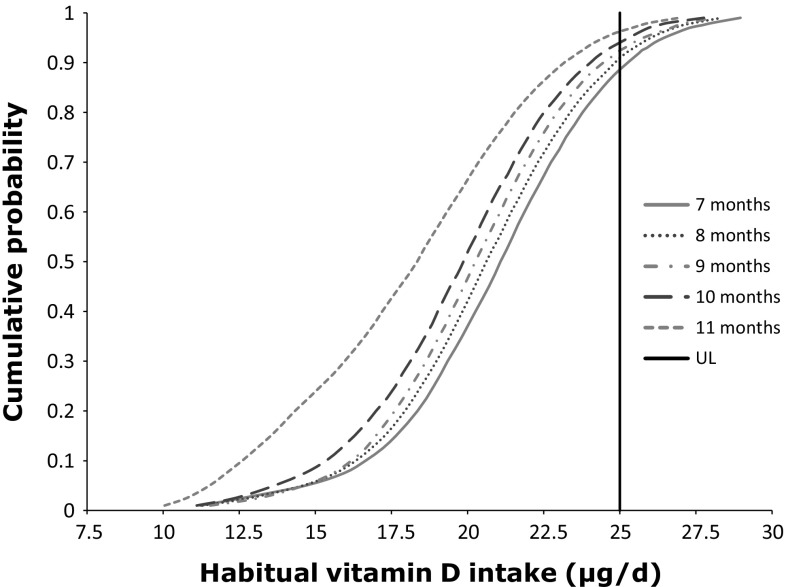
Habitual infant vitamin D intakes distribution (µg/day) from food and dietary supplements (as advised) and position towards the UL of 25 µg/day as set by EFSA [3]

In 2011, the median habitual vitamin D intake from foods only (including infant formula) decreased with age by about 75 %, from 10.9 µg/day for infants 7 months old to about 2.5 µg/day for infants 16–19 months of age (Table 5; Fig. 1). Logically, the habitual vitamin D intake increased with about 10 µg by the addition of the daily-advised dosage vitamin D from dietary supplements (Table 5). The habitual intake was modelled separately including and excluding a daily supplement of 10 µg; therefore, the observed increase was not exactly 10 µg due to modelling uncertainty.

**Table 5:** Habitual vitamin D intake (µg/day) distribution (5th, 50th, 95th percentile) from foods, including and excluding a daily supplement of 10 µg for infants aged 7–13 and 16–19 months in the Netherlands

Age (months)	UL (µg/day)	Habitual vitamin D intake (µg/day)					
		Food sources only			Food and daily supplement of 10 µg		
		P5	P50	P95	P5	P50	P95
7	25	4.9 (1.4–7.4)	10.9 (9.9–11.9)	16.7 (15.2–18.0)	14.7 (11.6–17.2)	21.1 (20.2–22.2)	26.5 (25.5–27.7)
8	25	4.9 (3.3–6.2)	10.5 (10.2–11.3)	16.2 (15.8–17.3)	14.6 (13.4–16.1)	20.6 (20.3–21.6)	26 (25.6–27.1)
9	25	5 (3.6–5.9)	10.1 (9.8–11.0)	16 (15.6–16.9)	14.6 (13.6–15.7)	20.3 (20–21.1)	25.8 (25.2–26.6)
10	25	3.7 (2.1–5.5)	9.6 (9.2–10.5)	15.8 (15.1–16.6)	13.7 (12.3–15.4)	19.9 (19.3–20.6)	25.3 (24.7–26.2)
11	25	1.3 (1.1–1.7)	8.3 (7.8–9.2)	14.8 (14.3–15.8)	11.5 (11.2–11.9)	18.3 (17.8–19.3)	24.5 (23.9–25.4)
12	50	0.8 (0.6–1.1)	5.7 (4.4–6.8)	13.5 (12.4–14.5)	10.7 (10.3–11)	15.4 (14.5–16.7)	23 (22.2–24.2)
13	50	0.8 (0.4–1.2)	4.6 (3.1–7.0)	12.6 (9.6–14.7)	10.6 (9.7–11.2)	14.6 (13.1–17.0)	22.1 (18.9–24.2)
16	50	0.5 (0.2–0.6)	2.4 (1.5–3.0)	9.7 (6.3–12.0)	9.6 (8.8–10.0)	13 (11.8–13.5)	19.1 (16.2–21.7)
17	50	0.5 (0.4–0.6)	2.6 (2.3–3.2)	9.8 (8.3–12.1)	9.7 (9.5–10.1)	13.1 (12.8–13.7)	19.1 (17.8–21.2)
18	50	0.5 (0.5–0.6)	2.6 (2.3–3.1)	9 (8.1–10.5)	9.7 (9.5–10.1)	13 (12.7–13.5)	18.4 (17.4–19.6)
19	50	0.5 (0.3–0.7)	2.5 (1.6–3.0)	8.5 (5.9–10.1)	9.7 (9–10.1)	12.9 (12–13.4)	17.6 (15.9–18.7)

Results are presented as point estimate and between brackets 95 % CI

#### Comparison with the UL

**Fig. 2:**
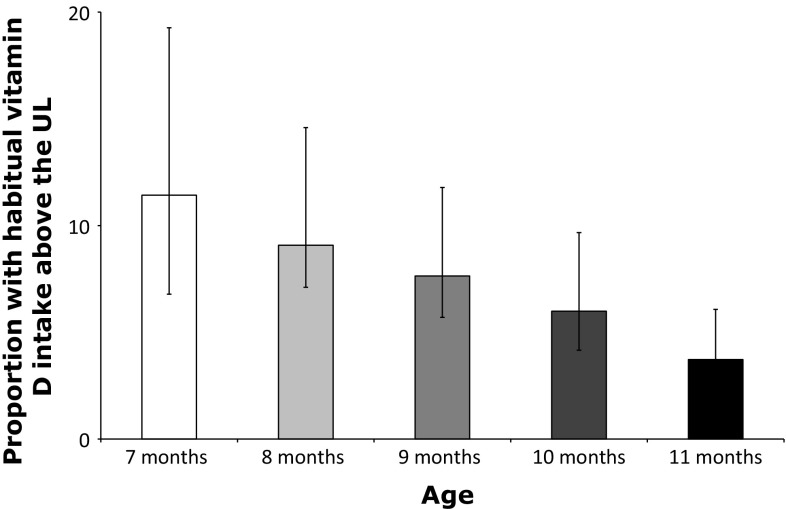
Proportion of infants (7–11 months of age) with vitamin D intakes from foods and the advised supplement dosage of 10 µg/day exceeding the UL of 25 µg/day [3]

The vitamin D intake from foods only remained below the UL for all ages (data not shown). Including a daily supplement containing 10 µg vitamin D, 4–11 % of the infants 7–11 months old had intakes exceeding the UL of 25 µg/day (Fig. 2). This percentage decreased with age. The vitamin D intake at the 99th percentile was 27–29 µg vitamin D per day for infants 7–11 months of age. For infants 12–13 and 16–19 months old, the total vitamin D intake remained below the UL of 50 µg/day.

## Discussion

The scenario study presented in this paper suggests that infants aged 0–6 months and 1 year and older are not at risk of exceeding the UL for vitamin D by combined intake from food and supplements (according to the supplement advice). Our study suggests, however, that some infants aged 7–11 months of age are at risk of exceeding the UL for vitamin D as set by EFSA by combined intake of vitamin D from regular food, infant formula, fortified foods, and supplements. Some of these infants still consume considerable amounts of infant formula sometimes combined with fortified foods. Together with following the current Dutch supplementation advice of 10 µg/day, this resulted in 4–11 % (decrease with age) of the infants with vitamin D intakes above the UL.

### Risk of adverse health effects

For children 7–11 months old, the vitamin D intake at the 99th percentile was 27–29 µg/day and decreased with age. This suggests that some infants are exceeding the UL at a limited time period of at maximum 5 months and with a limited amount (8–15 % above the UL) which decreased with age. From 1 year onwards, the UL is two times higher, namely 50 µg/day, and no exceedances were observed. The few studies that were used for setting the UL for infants (0–1 year) showed no adverse effects after a duration of about 4 months to 24 weeks [30]. This time period has a similar length as the period in our study at which some infants are at risk of vitamin D intakes above the UL.

An intake above the UL will, in most cases, not directly result in harmful health effects, as in setting the UL uncertainty factors are applied and the UL is generally set for health effects presenting after chronic high exposure. In addition, the risk of harmful health effects will be determined by the duration of high intakes and the actual amounts and varies from person to person [3, 31]. Moreover, the UL for infants is surrounded by uncertainties due to lack of data. This is illustrated by the higher ULs as set by IoM [2] compared to EFSA for children aged 7 months to 3 years. In our study, the 99th percentile of total vitamin D intake remained below the IoM ULs for all infants. Given the above arguments, the results of this study are not directly alarming, but do call for studies on the relationship between high vitamin D intakes during infancy and adverse health effects. In addition, we advise to be alert on potential adverse health effects that may be related to excessive vitamin D intakes in infants and monitor the habitual vitamin D intake regularly.

### Accuracy using label information for food composition

Other studies have shown that it is not uncommon that fortified foods contain higher amounts of micronutrients than declared on the package [32, 33]. This so-called overage is applied to ensure adequate levels of the nutrient at the end of the shelf life. We are aware of few published data regarding vitamin D overages in fortified foods or dietary supplements. In 1992, vitamin D overages were observed in infant formula in USA [34]. Seven of ten samples contained more than 200 % of the labelled amount. A more recent study in USA showed lower overage levels of 100–180 % of labelled values in infant formula [35]. In a New Zealand study, 12 of the 18 foods (i.e. baby food, food drink, margarine, milk product) had a measured vitamin D concentration that significantly deviated from the label claim [32]. For seven products, this was an overage. In the whole sample, the deviation ranged from −68 to +70 %. In a recent update of the vitamin D content of fortified foods and supplements in the UK Nutrition Survey Nutrient Databank, for each vitamin D level on the label a standard overage of 12.5 % was applied [36]. The overage at time of production was estimated to be on average 25 % (manufacturer and trade association: overages ranged 20–30 % for fortified foods and 20–40 % for supplements). The average remaining overage at time of consumption was assumed at 50 % of this 25 %. Taking into account, this overage of 12.5 % for all vitamin D fortified foods and supplements showed a 6 % increase in mean vitamin D intake in UK population aged 1.5 years and over. This proportion tended to be higher for the youngest age group (1.5–3 year), namely 9 %.

The importance of the fortified foods and supplements in the total vitamin D intake will influence the effect of potential overages on the intake. For infants in the Netherlands using vitamin D supplements as advised and in addition consuming fortified foods, the effect of overages on total intake might be substantial. If in this study the habitual total vitamin D intake from food and supplements will be increased due to overages by 9 % or even 12.5 % in children aged 7 months, the proportion with intakes above the UL would crudely be estimated at 30–35 % as compared to 11 % based on label information. However, even at high percentiles, the crude estimate of the habitual intake would be still below the UL of 37.5 µg/day as set by the IoM. It is recommended to study the overage (at time of consumption) in fortified foods and supplements, especially for products and/or nutrients contributing largely to the total nutrient intake.

### Intake from dietary supplements

In our scenario, all infants were assigned to a 100 % adherence to the vitamin D supplementation advice. Several studies showed that not all infants take supplements according to this advice [37–40]. In the Netherlands in 2012, the vitamin D supplementation advice for young children was adapted to make it unambiguous. In the new advice, all young children, despite the amounts of infant formula consumed, are advised to take a daily vitamin D supplement. There is no insight into the current vitamin D intake from supplements among children 0–4 year old. In a recent study among children visiting day care (at least 2 and maximum 5 days per week), about 90 % of the parents reported to provide vitamin D supplementation to their infant (10 months to 3 year) [41]. It was unclear whether dosages were provided and whether the supplement was given daily. The risk of exceeding the UL may be an overestimation if not all children use a vitamin D supplement daily, as it was shown that especially the combined intake from foods and supplements resulted in intakes above the UL. On the other hand, our estimate may be an underestimation if children take more vitamin D from supplements than advised [40]. Our study shows the potential risk of exceeding the UL if all children would follow the supplementation advice as is warranted in combination with available fortified foods in a realistic consumption pattern.

### Food consumption data

The food consumption data used in this scenario study are from 2002. The response rate was high (82.5 %), and the data can be considered representative for the Netherlands at that time. Total energy intakes of the infants indicated that there was no major problem of under- or overestimation at the group level [19]. It is possible that the consumption pattern of infants changed during the last decade; however, no recent data are available for the Netherlands. Also for other European countries, these data are scarce [42]. To get better insight into the food consumption of infants, it is recommended to conduct a food consumption survey regularly among this age group, preferably also including the younger ages 0–6 months and those consuming breast milk.

In our scenario study, for infants aged 0–6 months a crude estimate of vitamin D intake was made based on energy requirement and body weight. It was assumed that these infants did not consume any complementary foods; however, part of the infants younger than 6 months of age may already start consuming these foods [43]. The vitamin D intake estimated in our scenario study may be an underestimation for infants aged 3–6 months. On the other hand, the foods often started with, for example, vegetables and fruit generally do not contain large vitamin D levels. It is therefore expected that this will not affect the estimates of the vitamin D intake largely.

Originally, the VIO study was combined with Dutch food composition data from 2001. For our study, the food consumption data were combined with more recent Dutch food composition data from 2011, to reflect better the actual vitamin D intake. Based on a quick scan of the current market (2015) using similar resources as in 2011, it can be concluded that the current market of vitamin D fortified foods and supplements is comparable with the situation in 2011 (data not shown). As information was collected from several sources, we expect this inventory to be rather complete. For some foods, the highest level in a product group was applied what may lead to an overestimation. In general, the contribution of vitamin D fortified foods, other than infant formula, was low; therefore, the effect of this potential overestimation on the total vitamin D intake is considered minor. To improve the estimation of vitamin D intake, it is recommended to have an up-to-date overview of the vitamin D fortified foods available on the Dutch market including the vitamin D levels, for example, by notification.

The data of the VIO study were collected among a relative small study population in three specific age groups: about 9, 12, and 18 months. Consequently, these three ages are more represented in this study population than the surrounding ages. To get results per month of age, the habitual intake was not estimated separately per age group, but as a function of age incorporating the data of all ages. The higher uncertainty in results of the habitual intake distribution for age groups (months of age) with a small number of observations is taken into account in the 95 % confidence interval. As a result, the 95 % confidence intervals of age groups with a low *n* are generally wider. Although the number of children in some age months was rather low, a clear effect of age was observed.

### Scenario analyses

In this paper, we presented a scenario study. Scenario analyses are theoretical exercises only. But they provide insight into changes in the exposure distribution, where otherwise no rapid quantitative insight could be given. A number of assumptions are always needed to build scenarios, and it is important to interpret the results always in the light of these assumptions. The results are most predictive with realistic assumptions. From a policy making point of view, scenario analyses are very relevant as it may give at least some insight into what may the population impact of a (potential) change in policy. For instance, will the target population receive the adequate intake level with a specific fortification practice and is there no or limited risk of excessive intakes in the whole population. In addition, signals from scenario studies may be used to get the scope for additional research required to get more precise answers whether there is a health problem [44, 45].

A strength of this study was that the habitual total vitamin D intake from all potential vitamin D sources was estimated using a model that could cope with the multimodality of the data, namely a first-shrink-then-add approach. By splitting up the consumption data in vitamin D from infant/follow-on formula and vitamin D from other sources, two uni-modal distributions were created for which the habitual intake distribution could be estimated without violation of the model assumptions. It is important that the model assumptions are met, otherwise the habitual intake distribution may be estimated invalidly (especially at the tails), and as a result, the proportion at risk of excessive intakes may be invalid [24, 26]. This approach may be applied in research facing multimodal distributions which could be identified by splitting up the data in different sources of intake, such as voluntary fortification practices and intake from dietary supplements.

## Conclusion

In conclusion, infants aged 0–6 months and 1 year and older were not at risk of exceeding the UL. Some infants 7–11 months of age are at risk of vitamin D intakes above the UL with combined consumption from infant formula, (fortified) foods, and supplements. The largest risk is for 7-month-olds still consuming relatively large amounts of infant formula. It remains unclear whether a temporarily exceedance of the UL with a limited amount may cause harmful effects in infants at short or long term. Additional research is recommended for the association between high vitamin D intake and harmful health effects in infants. In addition, more insight is required in the actual vitamin D content of foods with added vitamin D and dietary supplements to study whether label information can be used for valid estimations of dietary intake or that correction for potential overages is required. Nonetheless, this partially virtual scenario study illustrates the possibility of vitamin D intake of older infants beyond the UL set by EFSA.

## Abbreviations

UL: Tolerable upper intake level
SPADE: Statistical Program to Assess Dietary Exposure
EFSA: European Food Safety Authority
IoM: Institute of Medicine
NethFIR: Netherlands Food Information Resource
NEVO: Dutch food composition database (in Dutch NEderlands VOedingsstoffenbestand)
VIO: Nutrient Intake Study (in Dutch: VoedingsstoffenInnameOnderzoek)
AI: Adequate intake
